# Airborne SARS-CoV2 virus exposure, interpersonal distance, face mask and perceived risk of infection

**DOI:** 10.1038/s41598-024-52711-2

**Published:** 2024-01-27

**Authors:** Ola Svenson, Freja Isohanni, Ilkka Salo, Torun Lindholm

**Affiliations:** 1 Department of Psychology, Stockholm University, Stockholm, Sweden; 2 Decision Research, Eugene, OR USA; 3 Department of Psychology, Lund University, Lund, Sweden

**Keywords:** Psychology, Health care, Medical research, Risk factors

## Abstract

Participants judged the risk of an infection during a face to face conversation at different interpersonal distances from a SARS-CoV-2 infected person who wore a face mask or not, and in the same questionnaire answered questions about Corona related issues. Keeping a distance to an infected person serves as a protective measure against an infection. When an infected person moves closer, risk of infection increases. Participants were aware of this fact, but underestimated the rate at which the risk of infection increases when getting closer to an infected person, e.g., from 1.5 to 0.5 m (perceived risk increase = 3.33 times higher, objective = 9.00 times higher). This is alarming because it means that people can take risks of infection that they are not aware of or want to take, when they approach another possibly virus infected person. Correspondingly, when an infected person moves away the speed of risk decrease was underestimated, meaning that people are not aware of how much safer they will be if they move away from an infected person. The perceived risk reducing effects of a face mask were approximately correct. Judgments of infection risk at different interpersonal distances (with or without a mask) were unrelated to how often a person used a mask, avoided others or canceled meetings during the COVID-19 pandemic. Greater worry in general and in particular over COVID-19, correlated positively with more protective behavior during the pandemic, but not with judgments of infection risk at different interpersonal distances. Participants with higher scores on a cognitive numeracy test judged mask efficiency more correctly, and women were more worried and risk avoiding than men. The results have implications for understanding behavior in a pandemic, and are relevant for risk communications about the steep increase in risk when approaching a person who may be infected with an airborne virus.

## Introduction

During the COVID-19 pandemic people were urged by health authorities to keep distance, wash their hands, wear a face mask and avoid gatherings of people. In the present work we will focus on keeping distance as a way to avoid infection. There are a number of studies of exposure and risk of infection when people are at different distances from an infected person^1–4^. However, there are almost no studies of how perceived exposure and risk depend on distance to an infected person. Because people’s behavior is governed by their perceptions, such studies are important for understanding behavior in a pandemic. The present authors are aware of only three studies focused on the problem of distance, exposure and perceived risk; two studies by Svenson et al.^5,6^, and one study by Heffertz and Rabin^7^. These studies showed that when a person approaches an infected person, the increase in exposure is underestimated because the participants used an incorrect exposure-distance function. These initial studies focused on exposure, and did not ask about perceived risk of catching COVID-19 as a function of distance to an infected person.

In the present study, we extended this line of research and investigated the subjective function between perceived risk of infection, and distance to a virus source and compared this function with the objective relationship based on research about dynamics of small particles in air^8–11^ as well as objective infection risks depending on exposure^12–15^. The relationship was investigated for a healthy person both with and without a face mask. We also studied relationships between judgments of risk of infection, attitudes and self-reported protective behavior during the pandemic.

Generally speaking, risk perception refers to various kinds of attitudes and judgments elicited by the awareness of a real risk of, e.g., catching a disease^16^. In the COVID-19 domain of risk perception research, Dryhurst et al. collected risk perceptions of COVID-19 from different countries during March and mid-April 2020^17^. They found that levels of concern were higher in the UK than in other parts of the world. Participants who had direct personal experience with the virus perceived the risk of infection as greater than those who had less experience, and greater magnitudes of perceived risk correlated positively with preventive health behaviors. Across the investigated countries, male participants perceived the risk of infection as smaller than women. Bish and Michie^18^ reviewed research on demographic and attitudinal determinants of protective behavior against the risk of infection during a pandemic. They found that the following factors affected protective behaviors, age (on average older persons were more protective), gender (women more protective behavior), perceived efficacy of protective behavior (higher efficacy more protective behavior), education (more educated more protective behavior), perceived susceptibility to the disease (greater susceptibility more protective behavior), perceived severity of disease (more severe more protective behavior), and perceived costs of protective behaviors (more real or imagined costs less protective behaviors). Research has also shown that worry and anxiety about a risk are important drivers of protective behaviors^19–22^. In the present study, we used these findings to formulate questions about attitudes and behavior that could be related to perceived infection risk at different distances from an infected person.

There are a number of studies of how people change interpersonal distances when one or both wear a face mask against the Corona virus^23–25^. However, none of these studies shows how a change of distance affects subjective appraisal of exposure or infection risks. But reversing the causality, Iachini et al.^26^ illustrated how people rely on their own anxiety and perception of virus exposure, and not on the actual risk, when they select safe interpersonal distances. Most of the studies comparing distances with and without a mask show that people prefer greater interpersonal distance without than with a mask^7^. In contrast, in a study of pedestrian behavior in Amsterdam, Liebst et al. found no differences in interpersonal distance between areas with an area based mandate to wear a mask compared with an area without the mandate^27^. However, we do not know how the pedestrians judged their own change in virus exposure with and without a mask in the different areas when they violated the social distance recommended at the time (1.5 m).

With the former studies in mind, it is time to turn to the objective function relating objective virus exposure to distance from the virus source. Following this, we will cite research on the relationship between virus exposure and risk of infection and finally present a model relating perceived risk to the distance to a virus source.

In a series of laboratory studies, Bjørn and Nielsen reported exposure to another person’s normal breathing in a calm laboratory face to face setting [^8^, Fig. 15]. The power function Exposure = 1.90 × Emission × Distance^−2.2^ describes their results. In another investigation by Nielsen et al.^19^ the exponent was − 2.3. Ball et al. studied airborne droplets exposure at different distances from a source with and without a face mask and the power function had the exponent = − 2.6 [^10^, Fig. 3]. Different kinds of face masks lowered the curve, while the exponent remained approximately the same and smaller than − 2.0. Melikov^11^ presented a review of studies with exposure as a function of distance^2,3,28–31^. The results were summarized by Melikov [^11^, Fig. 1] with a decreasing function that can be approximated by a power function with an exponent − 2 or smaller. This was also confirmed by other researchers^32^.

There is a positive correlation between a person's virus load and disease severity. The higher the virus exposure the greater probability of receiving a higher virus load^12–15^. Therefore, we assert that the average infection risk varies proportionally with virus exposure. There are four fundamental conditions that need to be fulfilled for rapid spread of respiratory viruses^33^: asymptomatic hosts, high viral load, stability of virus in the air, and strong binding affinity to human cells. Corona viruses meet these criteria and, in addition, there is a great variability in how contagious they are^34^. There are asymptomatic infected persons and a few superspreaders along with a majority of ordinary people acting as Corona virus sources. All generate different viral loads on exhaled droplets and aerosols. As mentioned above, virus exposure is a power function of distance with an exponent that is equal to or smaller than − 2.0. There is no reason to assume that this function changes with the number or characteristics of the virus traveling on the droplets and aerosols in an exhaust cloud. It is the characteristics and number of viruses that determine the risk of infection at each distance from the virus source along with the exposed person's own condition. In addition, the magnitude of the risk varies with the type of virus inhaled, e.g., SARS-CoV-2 virus and the Omikron type of the same virus with a higher infectivity^34^. However, it is possible to assume that change of distance from a source affects the number of airborne viruses inhaled by a person in a way that can be assumed to be proportional across different sources (e.g., superspreaders, the general population of infected people with normal spread of virus) and virus characteristics. This means that we assume the same exponent but different constants in the function.

Based on the empirical studies of airflow, airborne particles, and studies of objective risk of infection^2,3,8,10,19,28–32^, we created a model for risk of virus infection in Eq. (1). It is based on an earlier model for virus exposure presented by Svenson et al.^5^. Risk of virus infection, *Epv* is a function of virus emission *E,* distance to source *D*, and exposure time *t.* The exponent *n* describes the rate at which risk decreases with distance, and* a* is a constant. The model applies to both objective and perceived risk, but with different constants *a* and *n.*

1 $${\text{Epv}} = {\text{a}}*E*t*D^{ - n} \quad D > 0.5,\;t > 0$$

We called this model the Virus Emission Model, VEM and use *n* = 2.0 for objective risk. As mentioned earlier, it was clear from the studies cited above that this exponent, if anything, underestimates how fast objective exposure changes with changing distance, and we consider this to be the case also for objective risk of infection. This allows some margin on the conservative side. To illustrate, n = 2.0 predicts an increase in exposure for a person who approaches another infected person from 1.8 to 0.6 m, to be (1.8/0.6)^2^ = 9 times. However, a person who applies a linear model, n = 1.0 will judge the exposure to be 1.8/0.6 = 3 times the initial exposure after the approach. The study by Svenson et al.^5^ on perceived exposure showed that a number of participants used a linear model, joining the majority of the participants who underestimated the change of exposure following both approach and withdrawal from an infected person. It should be noted that the proportionality of exposure with time assumes a free ventilated space with no draft. For longer times, and poorly ventilated environments, the aerosols can accumulate and increase faster than linearly with time.

## Study

Even though the present study is primarily exploratory, we predicted (1) that the change in perceived Corona virus infection risk following a change of distance to a virus source will be smaller than objective estimates^5,6^. Based on a mental coherence assumption, we predicted (2) that the preferred minimum safety distance to an infected person will correlate with judgments of infection risk as a function of interpersonal distance. We also predicted, based on the studies cited above, that (3) more education will be correlated with less biased infection judgments, that (4) more worry will correlate positively with greater judged infection risk and more protective behavior, and (5) women will judge risk of infection as greater than men.

## Method

### Participants

200 participants aged 18 years or more were recruited by Prolific from a general UK adult English speaking population. The data were collected in May 2022. The number of participants was chosen with due regard to the results of two previous studies in the same project^5,6^ and the study was not preregistered. Two participants were excluded because of failure on an attention test, and 23 participants judged infection risk as increasing with increasing distance for a majority of the scenarios in the no mask condition and were excluded in all conditions, leaving 175 participants for further analyses. The mean age of the remaining participants was 45.29 (SD = 13.49). The median age 44.00 years is a little higher than the median age of UK citizens, which was 40.70 years in 2021^35^. There were 126 women, 48 men and 1 person with unspecified gender. 69 participants had been diagnosed with COVID-19 but they did not differ from the other participants on any variable analyzed in the present study. 27 participants had GCSE (general certificate of secondary education 2 years) level education, 26 had A-Level (secondary level 4 years) education, 8 Undergraduate education (e.g., University examinations but not completed degree), 79 Degree or Graduate education (e.g. BSc, BA), 21 Master’s degree, 4 Doctorate or PhD and 10 Vocational education. The purpose of the study and the possibility of leaving the study whenever a participant wanted, were presented before the study. Informed consent was obtained from all individual participants included in the study. The procedures used in this study adhere to the tenets of the Declaration of Helsinki and the University of Stockholm ethical guidelines.

### Procedure and scenarios

A Qualtrics questionnaire with scenarios and questions was distributed to the participants. On average a participant used 14 min to complete the task, and the instruction started with “*As you know, the Corona virus spreads on small droplets and aerosols (very small airborne particles) in the air when a person infected with the virus breaths, coughs, sneezes or talks. Therefore, keeping a physical social distance and using a face mask both reduce virus exposure and the risk of the virus to spread from person to person. We will ask you to judge how different distances and/or a face mask can change your exposure to the virus and risk of infection, when we assume that you do not wear a mask yourself in a face to face conversation with a person infected with the SARS-CoV-2 Omicron virus and sick with COVID-19*.”

The scenarios concerned relationships between risk of infection and distance to another person in a face to face conversation, and the instruction to one of the scenarios included “…*Assume that you are not vaccinated and standing face to face with a Corona virus infected person (also without a mask) and talk for 1 min. You stand 0.5 m from each other in a well ventilated environment with no draft. On average 100 of 1000 healthy unvaccinated persons are infected with the virus in this situation. If the same conversation takes place at different interpersonal distances, how many of 1000 healthy persons on average would be infected and sick with COVID-19 when the interpersonal distance is: 1.0 m? ___ 1.5 m?___ 2.0 m?___*.”

These scenarios were presented once again, this time with the order of distances reversed. Each scenario presented the distances at the same time to enable the participants to express their view on the curvature of the function coupling perceived risk to distance. Also, the presentations were analogue to an approaching and moving away context. The scenarios were repeated with the infected person wearing "*an ordinary commercial face mask stopping 60% of exhaled particles"* and with a person wearing *"a FFP2 mask stopping 95% of exhaled particles".* We allowed some leakage of the latter mask, which in a laboratory test context should stop 99% of particles. The scenarios were presented in a balanced order, so that half of the participants started with an increasing distance set of scenarios and half of the participants with a decreasing distance set of scenarios. The scenarios without a mask were always presented first, followed by scenarios with a commercial mask and a FFP2 mask in that order. Each numerical response was typed into a box in the questionnaire. The no mask condition had distances 0.5–2.0 m and the mask conditions 0.5–1.5 m, in avoidance of risks so small that they would be ignored and impossible to estimate by most people. A set of questions followed and asked for a participant’s behaviors, knowledge and attitudes related to Corona virus infection during the COVID-19 pandemic. Finally, we added some questions measuring numeric ability by a combination of 3 items from The Berlin Numeracy Test^36,37^ and 3 items from the Cognitive Reflection Test^38^. This combination of items has been used previously by other researchers to measure cognitive numeracy ability^39^. The questions and the scenarios in the test can be found in Supplement 1.

### Ethics approval

The procedure used in this study was approved by the Ethic guidelines of the Office for Research, Engagement and Innovation Services at Stockholm University and adheres to the Helsinki declaration.

### Consent to participate

The purpose of the study and the possibility of leaving the study whenever a participant wanted were presented before the study. Informed consent was obtained from all individual participants included in the study.

## Results

### Perceived infection risk, interpersonal distance and face mask

For each participant the judgments for a distance in the increasing and decreasing series of presentations were first averaged, because there were no significant differences between the judgments depending on order of presentation. The raw data can be found in Supplement 2. The means were used in the following data analyses. Table 1 shows the medians, 25th and 75th percentiles of judgments of number of people estimated to be infected out of 1000 persons. If an average judgment was greater than the reference 100, we decided to treat that value as missing, and in this way 25 (2%) judgments across scenarios and conditions were excluded as clear from the table. The table also shows the number of infected persons predicted by the VEM model with the exponent = − 2.0 and a linear proportional model.

**Table 1:** Judged number of exposed persons that are infected and values predicted by the VEM model with the exponent = − 2.00. The instruction specified that at the comparison distance 0.5 m without a mask, 100 out of 1000 persons were infected.

	Median 25th–75th percentiles	Mode	N	Linear proportional prediction	VEM predictions	Median—VEM
Condition no mask						
Reference value Dist = 0.5 m	100.00		–	100.00	100.00	
Dist = 1.0 m	50.00 (50.00–62.75)	50.00	165	50.00	25.00	25.00***
Dist = 1.5 m	30.00 (25.00–50.00)	25.00	168	33.00	11.11	17.65***
Dist = 2.0 m	13.30 (10.00–25.00)	25.00	172	25.00	6.25	7.05***
Condition commercial mask						
Reference value Dist = 0.5 m	40.00		–	40.00	40.00	
0.5 m	40.00 (40.00–61.30)	40.00	162	40.00	40.00	0.00
1.0 m	25.00 (20.00–40.00)	20.00	165	20.00	10.00	15.00***
1.5 m	15.00 (8.75–27.50)	10.00	169	13.00	4.44	10.56***
Condition FFP2 mask						
Reference value Dist = 0.5 m	5.00		–	5.00	5.00	
0.5 m	5.00 (5.00–20.00)	5.00	170	5.00	5.00	0.00
1.0 m	3.50 (2.00–10.00)	2.00	170	2.50	1.25	2.25***
1.5 m	2.00 (1.00–5.00)	1.00	172	0.80	0.56	1.45***

***Difference from VEM prediction, alpha = 0.001 Wilcoxon rank test.

In the no mask condition, the average remaining median risk was always significantly greater than predicted by VEM, which supports our first prediction. Overall, more than 75% of the participants made judgments that were greater than those predicted by VEM. We used VEM and a mask's filtering capacity to predict the risks at different distances. In the commercial mask and FFP2 conditions, the median and mode indicated that most participants correctly judged the stated filtering capacity of the masks at 0.5 m, but when the distance increased, more than 75% were not aware of the strong decline in risk with increasing distance. Table 1 also tells us that the participants always underestimated the increase in risk when coming closer to an infected person. To exemplify, a comparison of the judged risk of infection at 1.5 m (30.00) with the standard risk at 0.5 m (100.00) shows that the perceived increase in risk was only 100/30 = 3.33 times, but the objective risk increase is 100.00/11.11 = 9.00 times. This supports our first prediction. The results are alarming because they mean that people can take risks that they are not aware of or want to take, when they approach another possibly virus infected person.

Across conditions, all judgments in Table 1 were significantly greater than predicted by VEM, and they seemed to approximate a linear proportional rule rather than a power function with the exponent equal or smaller than − 2.0. Hence, it is probable that the risk estimates in the present study were influenced by cognitive processes favoring linear over curved functions^37,38^.

Comparisons of the no mask condition with the commercial mask condition for 1.0 and 1.5 m give (50.00 − 25.00)/50.00 = 50% and (30.00 − 15.00)/30.00 = 50% risk reduction with the commercial mask. The corresponding numbers for the FFP2 mask are (50.00 − 3.50)/50.00 = 93% and (30.00 − 2.00)/30.00 = 93%. These numbers can be compared with the objective 60% and 95% reductions of the exposure specified for the masks and show that the participants were adjusting their judgments to the filtering capacities of the masks well and in particular for the most effective mask. Interestingly, this also shows that the underestimation of the protective effect of distancing was invariant across conditions.

### Corona virus understanding, attitudes and behavior

We created an index of a person’s overall judgment of infection risk depending on distance by computing the mean judgment for each of the conditions (no mask, commercial, FFP2 mask). This gives a *distancing infection index* and a measure of a person’s overall perception of infection risk, depending on distance for each of the conditions. The smaller this value the more efficient the increase in distance as a mean of reducing infection risk. The Corona related items are referred to in the text by a letter and a number and listed in Table 2. The exact wordings of the items can be found in Supplement 1.

**Table 2:** Principal components analysis of Corona virus related attitudes, behavior and protective effects of changing distance to an infected person. Factor loadings greater than 0.70 italicized.

	Worry and own protective behavior	Effect of following recommendations	Effect of distancing	Cognitive aptitude
Cognitive numeracy test				*0.82*
Level of education				0.44
Distance no virus G1			0.43	
Shortest safe distance no mask virus G3			0.47	
Own worry sick with COVID-19 G5	*0.78*			
Own worry in general G6	0.58			− 0.38
Effect of distancing during pandemic G7		*0.82*		
Effect of 60% mask during pandemic G8		*0.89*		
Effect distance and mask during pandemic G9		*0.89*		
Likely own infection COVID-19 G12	0.44			
Severity of COVID-19 G13	0.51	0.31		
Inconvenience follow advise G14	− 0.37			
Used face mask in public transports G16	0.56	0.45		
Avoided people during pandemic G17	*0.78*			
Cancelled meetings G18	*0.80*			
Judged effect of change of distance no mask			*0.74*	
Judged effect of change of distance 60% mask			*0.78*	− 0.31
Judged effect of change of distance 95% mask			0.64	− 0.50

Component analysis, varimax rotation, 54% explained variance.

The participants indicated the *shortest safe distance* to a person with a COVID-19 infection that would make them feel safe from becoming infected, (G3), mean = 3.02 (SD = 4.18) meters. This is longer than the mean safe distance in a Corona context hovering around 1.70–1.85 m, obtained by Welsch et al. during the pandemic^48^. They used a male graphical silhouette to measure the distance. The shortest safe distance variable was correlated with the distancing index for each of the conditions, no mask, commercial mask and 95% mask. The results were R (175) = 0.23, p < 0.01, R (175) = 0.22, p < 0.01 and R (175) = 0.20, p < 0.01. This means that if a person’s perceived reduction of infection risk was relatively smaller in a condition (greater means), their required safe distance was relatively larger, which is cognitively coherent, and supports our second prediction.

An index of the results on the cognitive numerical problems was calculated by giving a correct answer = 1 and an incorrect answer = 0, and then summing across the problems. Cronbach’s alpha was 0.73 for the set of problems. This index was correlated with the distance infection index in the different conditions, R (175) = 0.04, non significant, for the no mask condition, and R (175) = − 0.19, p < 0 0.05 and R (175) = − 0. 21, p < 0.01 for the commercial mask and FPP2 mask conditions respectively. Thus, higher cognitive numerical ability was associated with lower judgments of infection risk values in the mask conditions, indicating greater understanding of the protective effects of distance with masks. *Education* was not correlated significantly with judgments of the effects of distance and mask protection on infection risk and therefore our third prediction was not supported.

The participants judged their use of *face mask* (G16), *avoiding public spaces* (G17) and *postponed/canceled meetings* (G18) as rather high with means 89.40 (21.50), 75.90 (29.50) and 69.70 (34.40) on a response scale ranging from 0 (never) to 100 (always). *Age* and one of the social protective behavior items *avoiding gatherings* (G17) correlated significantly R(175) = 0.18, p < 0.05 meaning that older people avoided gatherings more often than younger persons.

To get an overview of the items covering attitudes and behavior related to the Corona virus and the distancing infection indices, we used a principal components analysis which explained 54% of the variance. Because of the strong effect of worry on behavior found in previous studies^19–22^, we expected a strong worry component. We also expected one component for distance scenarios including distance behavior and efficiency of distancing during the pandemic. The result of the analysis gave the following factors “Worry and own protective behavior”, “Effect of following recommendations”, “Effect of distancing” and “Cognitive aptitude” and Table 2 shows the results.

The “Worry and own protective behavior” factor included *worry COVID-19* and own *worry in general*, but also social behavior during the pandemic, *avoided people* and *canceled meetings.* This factor had loadings, albeit smaller loadings, on *severity of COVID-19* and *use of face masks.* This supports our fourth prediction concerning the role played by worry.

The “Effects of following recommendations” factor was characterized by high loading on the risk reducing effects of always *following behavioral recommendations,* and it also had a modest loading on *use of face mask* in public transports. The “Effect of distancing” factor included lower loadings on a person’s distance during a conversation both with an infected person and with a healthy person.

It was surprising and informative to find that the effects of distancing during the pandemic was included in factors other than the judgments of risk of infection at different distances. The last factor, “Cognitive aptitude” was high on the *Cognitive numeracy test,* with only smaller loadings on *Education* and the two items about effects of change of distance on risk with a mask.

We repeated the component analysis with men and women separately, and found the same factor structure. In spite of the structural similarities, there were some differences between the genders on some of the items. All judgment scales reached from 1 to 100 for all items. For *own worry to get sick*, the mean for women was 57.60 (SD = 31.70) and for men 43:00 (SD = 34.10), t (173) = 2.65 p < 0.01, Cohen’s d = 0.44. This supports the fifth of our predictions. For *worry in general,* the mean for women was 48.6 (SD = 27.00) and for men, mean = 38.3 (SD = 26.60), t(173) = 2.25 p < 0.01, Cohen’s d = 0.38. Women *avoided people*, mean = 79.80 (SD = 26.40) to a greater extent than men, mean = 65.80 (SD = 34.80), t (173) = 2.86 p < 0.01, Cohen’s d = 0.45, and found *following advice* from authorities to be less inconvenient, mean = 42.00 (SD = 32.6) than men, mean = 55.30 (SD = 34.90), t = 2.86 p < 0.01, Cohen’s d = 0.39. It was interesting to find that men were less worried than average on the scale and that women were more worried than the average, which may support the findings by Dolinsky et al.^49^. However, we did not find any general optimism effect^50–52^ when we asked “Assume a new Coronavirus epidemic occurred and no vaccine was available. Compared to the average person like yourself, how likely do think that it is that you would become sick?” In summary, women were more worried, and took less risks than men, in line with previous work^40^.

The correlation between *shortest safe distance* and judgments of the efficiency of keeping *distance* as a protection against infection differed between the genders with R (125) = − 0.02, ns. in the female group and R(48) = 0.33, p < 0.05 in the male group. The difference between the correlation coefficients was significant and, given the principal component analysis covering all participants, it was not surprising to find no coupling between minimum *shortest safe distance* and perceived effect of *distancing* in the female group, because it was much bigger than the male group of participants.

## Discussion

Risks are often described in terms of probability of a negative event or outcome. In the present study we focused on the probability part of the risk of an infection. When an objective risk is observed by a person, the subjective representation of that risk is called risk perception. Creation and elicitation of risk perception can be described as a sub-process in a mental model covering a specific domain^41^.

This process includes integration of the available information and causal chains^42–44^. The present paper concerns a dose response problem, in which the dose is the exposure, and the response is the perceived risk. The relation describing perceived risk of infection as a function of distance was the same as the function relating exposure to distance in other studies^5,6^. Therefore, perceived risk of infection can be assumed to be proportional to judged exposure, which is also found for objective measures in medical research treating virus exposure and risk of infection^12–15^.

We found that a great majority of the participants used the wrong function, often closer to a linear proportional model than a power function, when distance from an infected person decreased (risk increases). The incorrect function made them underestimate the increase of risk in an approach to an infected person. This can lead to unintended risk taking when a person approaches an infected person (objective risk increases faster than perceived risk). In other words, the person may think that the risk is smaller than it is objectively, and at a level that she or he would not accept if correctly informed.

This finding should be addressed in future risk communications. Those higher in cognitive aptitude seemed to have a more correct mental model, and made significantly less biased judgments of infection risk. It was interesting to find that the virus protective efficiency of masks were realistic across different distances to an infected person. Hence, the existing format of information about the efficiency of masks can be understood and used by people, and therefore it can be used, in its present format, for risk communications in the future. The insensitivity to the change of exposure with decreasing distance also applies to movements away from an infected person^5,6^. Therefore, people are not aware of how fast risk decreases in a movement away from an infected person. This may lead to neglect of a necessary withdrawal—“risk seeking” behavior (“just withdrawal a short distance will not make much difference in risk, so I do not bother to move away”). Or it may lead to a withdrawal further away decreasing real risk more than intended—“risk avoidance” behavior (“moving to be sure”).

When people make judgments of risks like the ones made in the present study, there is no perceptual, experiential, or cognitive feedback available as, for example, when you throw a ball and see that the ball follows a parabolic trajectory, which you can learn to handle. When there is no feedback, simpler linear, or proportional relationships among variables are activated before curved functions are attempted^43–47^ and this seems to be the case for many participants in the present study.

It was interesting to find that the principal component analysis indicated that worry and protective behavior were closely linked but unrelated to the judged efficiency of following recommendations. One would assume that those who believed more strongly in the protective effects of following recommendations also should be more eager to follow them, but this was not the case. It is possible to assume that those who behaved more cautiously would also be more aware of the protective effects of different distances to an infected person. However, the effects of different degrees of distancing with or without a mask were unrelated to how often the participants used masks, avoided others or canceled meetings. This may be described as if the participants had one “mental account” for the effect of the *length of the distance* in each distancing episode, and another for the effect of *frequency of distancing episodes* in their daily life. These mental accounts seem to have just one connection, a person's judged minimum safe distance to an infected person during a conversation.

The present study has possible limitations. The participants were UK citizens, and even though they know the meter system, some of them may still think in feet and inches most of the time. However, uncertainty about exactly how long a meter is does not influence the result here, because we use ratios of distances and the functions also use ratios of distances. Another possible limitation concerns the numerical responses and the participants who were excluded because they gave risk responses below 100 in an approach scenario. This may indicate a problem with using the numerical response scale and a systematic exclusion of participants who were less numerically skilled or motivated. Hence, numerically relatively more advanced participants may be over-represented in our analyses. However, the study reports systematically biased judgments, therefore the biases should be even greater if the excluded participants had been included in the analyses. There may also be participants who had a difficulty in understanding the scenarios correctly in the excluded group. Finally, the use of a questionnaire is always coupled with a limitation concerning participant motivation and generalizations to actual behavior. This latter problem should be addressed in future studies. Given the results of the present study, what information should be provided in a risk communication about the Corona virus and the risk of getting COVID-19? First, a risk communication should inform about the insensitivity to change of risk when moving closer to an infected person. This can be done by presenting a series of numbers of risks at different distances and/or with pictorial presentations of a person on a distance scale at different distances from a virus source and the risk at the distance shown in each new pictorial presentation. A pictorial representation was used successfully in a study of perceived exposure as a function of distance^7^. The effects of mask on risk reduction can be communicated using the ordinary mask specifications.

In conclusion, we found (1) systematic underestimation of the increase in infection risk when a healthy person approaches a Corona virus infected person, (2) that the participants understood information about face mask filtering efficiency and adjusted their risk judgments accordingly, (3) that the subjective function describing perceived risk as a function of distance to a virus source was not related to attitudes, self-reported distancing and other protective behavior during the COVID-19 pandemic, (4) that in risk communications, pictorial representation of distance combined with numerical information should be recommended.

### Supplementary Information

Supplementary Information 1.

Supplementary Information 2.

## Data Availability

The raw data can be found in Supplement file 2: *Svenson *et al*. Airborne SARS-CoV-2.csv.*

## References

[1] Chu DK, Akl EA, Duda S, Solo K, Yaacoub S, Schünemann HJ (2020). Physical distancing, face masks, and eye protection to prevent person-to-person transmission of SARS-CoV-2 and COVID-19: A systematic review and meta-analysis. Lancet.

[2] Bolashikov ZD, Melikov AK, Kierat W (2012). Exposure of health care workers and occupants to coughed airborne pathogens in a double-bed hospital patient room with overhead mixing ventilation. HVAC R. Res..

[3] Olmedo I, Nielsen PV, Ruiz de Adana M, Jensen RL (2013). The risk of airborne cross infection in a room with vertical low-velocity ventilation. Indoor Air.

[4] Fu L, Nielsen PV, Wang Y, Liu L (2022). Measuring interpersonal transmission of expiratory droplet nuclei in close proximity. Indoor Built. Environ..

[5] Svenson O, Appelbom S, Mayorga M, LindholmÖjmyr T (2020). Without a mask: Judgments of Corona virus exposure as a function of inter-personal distance. Judgm. Decis. Making.

[6] Svenson O (2022). Perceived Corona virus exposure as a function of interpersonal distance and time of a conversation. Discover Soc. Sci. Health..

[7] Heffetz O, Rabin M (2023). Estimating perceptions of the relative COVID risk of different social-distancing behaviors from respondents’ pairwise assessments. Proc. Nation. Acad. Sci.

[8] Bjørn E, Nielsen PV (2002). Dispersal of exhaled air and personal exposure in displacement ventilated rooms. Indoor Air.

[9] Nielsen PV, Olmedo I, de Adana MR, Grzelecki P, Jensen RL (2012). Airborne cross-infection risk between two people standing in surroundings with a vertical temperature gradient. HVAC&R Res..

[10] Ball L, Alberti S, Belfortini C, Almondo C, Robba C, Battaglini D (2021). Effects of distancing and pattern of breathing on the filtering capability of commercial and custom-made facial masks: An in-vitro study. PLoS ONE.

[11] Melikov AK (2020). COVID-19: Reduction of airborne transmission needs paradigm shift in ventilation. Build. Environ..

[12] Armstrong TW, Haas CN (2007). A quantitative microbial risk assessment model for Legionnaires’ disease: animal model selection and dose response modeling. Risk Anal..

[13] Rao SN, Manissero D, Steele VR, Pareja J (2020). A systematic review of the clinical utility of cycle threshold values in the context of COVID-19. Infect. Diseas. Therapy.

[14] Riley EC, Murphy G, Riley RL (1978). Airborne spread of measles in a suburban elementary school. Am. J. Epidem..

[15] Sze T, Chao CYH (2010). Review and comparison between the Wells-Riley and dose-response approaches to risk assessment of infectious respiratory diseases. Indoor Air.

[16] Slovic, P. History. in (P. Slovic, Ed.) *The Perception of Risk*, 21 -37*.* (Earthscan, 2000).

[17] Dryhurst, S., Schneider, C. R., Kerr, J., Freeman, A. L., Recchia, G., Van Der Bles, A. M., *et al*. Risk percept of COVID-19 around the world. in *COVID-19*, 162–174. (Routledge, 2022).

[18] Bish A, Michie S (2010). Demographic and attitudinal determinants of protective behaviours during a pandemic: A review. Br. J. Health Psychol..

[19] BruinedeBruin WB, Bennett D (2020). Relationships between initial COVID-19 risk perceptions and protective health behaviors: A national survey. Am. J. Prev. Medic..

[20] Frounfelker RL, Santavicca T, Li ZY, Miconi D, Venkatesh V, Rousseau C (2021). COVID-19. Experiences and social distancing: Insights from the theory of planned behavior. Am. J. Health Promot..

[21] Shiloh S, Peleg S, Nudelman G (2021). Adherence to COVID-19 protective behaviors: A matter of cognition or emotion?. Health Psychol..

[22] Vacondio M, Priolo G, Dickert S, Bonini N (2021). Worry, perceived threat and media communication as predictors of self-protective behaviors during the COVID-19 outbreak in Europe. Front. Psychol..

[23] Cartaud A, Quesque F, Coello Y (2020). Wearing a face mask against COVID-19 results in a reduction of social distancing. Plos One.

[24] Kroczek LO, Böhme S, Mühlberger A (2022). Face masks reduce interpersonal distance in virtual reality. Sci. Rep..

[25] Luckman A, Zeitoun H, Isoni A, Loomes G, Vlaev I, Powdthavee N, Read D (2021). Risk compensation during COVID-19: The impact of face mask usage on social distancing. J. Exp. Psychol. Appl..

[26] Iachini T, Frassinetti F, Ruotolo F, Sbordone FL, Ferrara A, Arioli M (2021). Social distance during the COVID-19 pandemic reflects perceived rather than actual risk. Int. J. Environ. Res. Publ. Health.

[27] Liebst LS, Ejbye-Ernst P, de Bruin M, Thomas J, Lindegaard MR (2022). No evidence that mask-wearing in public places elicits risk compensation behavior during the COVID-19 pandemic. Sci. Rep..

[28] Ai ZT, Hashimoto K, Melikov AK (2019). Influence of pulmonary ventilation rate and breathing cycle period on the risk of cross-infection. Indoor Air.

[29] Liu L, Li Y, Nielsen PV (2016). Short-range airborne transmission of expiratory droplets between two people. Indoor Air.

[30] Olmedo I, Nielsen PV, Ruiz de Adana M (2012). Distribution of exhaled contaminants and personal exposure in a room using three different air distribution strategies. Indoor Air.

[31] Villafruela JM, Olmedo I, San Jose JF (2016). Influence of human breathing modes on airborne cross infection risk. Build. Environ..

[32] Wang Y, Xu G, Huang Y-W (2020). Modelling the load of SARS-CoV-2 virus in human expelled particles during coughing and speaking. Plos One..

[33] Lee BU (2021). Why does the SARS-CoV-2 Delta VOC spread so rapidly? Universal conditions for the rapid spread of respiratory viruses, minimum viral loads for viral aerosol generation, effects of vaccination on viral aerosol generation, and viral aerosol clouds. Int. J. Environ. Res. Publ. Health.

[34] Riediker, M., Briceno-Ayala, L., Ichihara, G., Albani, D., Poffet, D., Tsai, D. H., *et al*. Higher viral load and infectivity increase risk of aerosol transmission for Delta and Omicron variants of SARS-CoV-2. *Swiss Med. Weekly*. 2022, 152.w30133 (2022). 10.4414/smw.2022.w30133 35019196

[35] Office for National Statistics People, Population and Community. (2021). https://www.ons.gov.uk/peoplepopulationandcommunity/populationandmigration/populationestimates/bulletins/annualmidyearpopulationestimates/mid2021

[36] Schwartz LM, Woloshin S, Black WC, Welch HG (1997). The role of numeracy in understanding the benefit of screening mammography. Ann. Internal. Med..

[37] Cokely ET, Galesic M, Schulz E, Ghazal S, Garcia-Retamero R (2012). Measuring risk literacy: The Berlin numeracy test. Judgm. Dec. Making.

[38] Frederick S (2005). Cognitive reflection and decision making. J. Econom. Perspect..

[39] Lind T, Erlandsson A, Västfjäll D, Tinghög G (2018). Motivated reasoning when assessing the effects of refugee intake. Behav. Publ. Policy.

[40] Slovic, P. Trust, emotion, sex, politics and science: Surveying the risk-assessment battlefield. in (P. Slovic, Ed.) *The Perception of Risk*, 390–412*.* (Earthscan, 2000). 10.1023/a:1007041821623 10765431

[41] Morgan MG, Fischhoff B, Bostrom A, Atman CJ (2002). Risk Communication: A Mental Models Approach.

[42] Svenson O (1985). (1985) Cognitive strategies in a complex judgment task: Analysis of concurrent verbal reports and judgments of cumulated risk over different exposure times. Org. Behav. Hum. Decis. Proc..

[43] Rieskamp J, Otto PE (2006). SSL: A theory of how people learn to select strategies. J. Exp. Psychol. General.

[44] Brehmer B (1971). Subjects’ ability to use functional rules. Psychon. Sci..

[45] Svenson O (2016). Towards a framework for human judgements of quantitative information: The numerical judgment process, NJP Model. J. Cogn. Psychol..

[46] Bitterly TB, VanEpps EM, Schweitzer ME (2022). The predictive power of exponential numeracy. J. Exp. Social Psychol..

[47] Lindskog M, Winman A, Juslin P (2013). Naïve point estimation. J. Exp. Psychol. Learn. Mem. Cognit..

[48] Welsch R, Wessels M, Bernhard C, Thönes S, von Castell C (2021). Physical distancing and the perception of interpersonal distance in the COVID-19 crisis. Sci. Rep..

[49] Dolinski D, Dolinska B, Zmaczynska-Witek B, Banach M, Kulesza W (2020). Unrealistic optimism in the time of coronavirus pandemic: May it help to kill, if so—Whom: Disease or the person?. J. Clin. Med..

[50] Weinstein ND (1980). Unrealistic optimism about future life events. J. Person. Social Psych..

[51] Svenson O (1980). Are we all less risky and more skillful than our fellow drivers?. Acta Psychol..

[52] Zell E, Strickhouser JE, Sedikides C, Alicke MD (2020). The better-than-average effect in comparative self-evaluation: A comprehensive review and meta-analysis. Psychol. Bull..

