# Supplementary material for: Airborne SARS-CoV2 virus exposure, interpersonal distance, face mask and perceived risk of infection
Source: Sci Rep. 2024 Jan 27;14:2285. doi: 10.1038/s41598-024-52711-2 (PMC10821858; doi:10.1038/s41598-024-52711-2)
Supplement: Supplementary file 1 — Supplementary Information 1. [file 41598_2024_52711_MOESM1_ESM.docx]

**Airborne SARS-CoV2 virus exposure, interpersonal distance, face mask and perceived risk of infection**

Ola Svenson ^1,2^, Freja Isohanni ^1 ,^ Ilkka Salo ^3^ , and Torun Lindholm^1^

**Questionnaire and cognitive test items**

G1. You have answered questions about inter personal distance, face masks and virus exposure.
 What average distance do you keep to a person in a normal face to face conversation when no virus is around? Please use decimals.

G3. What was the shortest distance between you and a Corona virus infected person without a mask that would make you feel sufficiently safe to start a conversation of 5 minutes during the last wave of the COVID-19 pandemic?

G 5. How worried were you over your own personal risk of becoming sick with COVID-19 during the last wave of the COVID-19 pandemic?
(0 = not at all, 100 = Maximum)

G 6. In general, how worried are you over things that may go wrong in your life?
 (0 = Not at all, 100 = Maximum)

G 7. If you always followed the advise of keeping distance to other people. What is the probability that this behavior could protect you from being infected by a Corona virus?
(0 = Not at all, 100 = Completely certain)

G 8. If you always followed the advise of keeping wearing a commercial 60% mask. What is the probability that this behavior could protect you from being infected by a Corona virus ?
 (0 = Not at all, 100 = Completely certain)

G 9. If you always followed the advise of keeping distance to other people and wearing a commercial 60% mask. What is the probability that this behavior could protect you from being infected by a Corona virus ? (0 = Not at all, 100 = Completely certain)

G 10. Have you been diagnosed with COVID-19 by a test or by a doctor ?

G 11.How many times have you been vaccinated against COVID-19?

G 12. Assume a new Coronavirus epidemic occurred and no vaccine was available. Compared to the average person like yourself, how likely do think that it is that you would become sick ?
(Much less risk = 1, same risk as average person = 50, much greater risk =100)

G 13. Compared with a normal seasonal flu, how severe was the Covid- 19 disease?
(0 = Not at all more severe, 100 = Maximally more severe)

G 14. How inconvenient was it to follow all official advise for protection against the Corona virus and the Covid-19 disease?
 (0= No inconvenience at all, 100 = Maximum inconvenience)

***Which of the following have you done during the last wave of the Corona virus pandemic?***

G 16. Did you wear a face mask when you moved in public spaces or transports?
 (0 = Never, 100 = Always)

G 17. Avoided public spaces, gatherings or crowds  more often than before the pandemic?
 (0 = Never, 100 = Always)

G 18. Canceled or postponed meetings with friends more often than before the pandemic?
(0 = Never, 100 = Always)

***The cognitive numeracy test consisted of the following items***

> In a small American lottery the chance of winning 10 dollars is 1%. How many do you guess will win 10 dollars if 1000 persons buy one ticket each? *( 10 correct answer )*

> Imagine that we throw a five-sided die 50 times. How many of these 50 throws will this die eventually land on an uneven number (1, 3 or 5)? *( 30 correct answer)*

> Out of 1000 people in a town, 500 are members of a choir. Out of these 500 choir-members, 100 are men. Out of the 500 people not members of a choir, 300 are men. What is the probability that a randomly drawn man is a member of a choir? *( 25% correct answer)*

>If it takes 5 machines 5 minutes to make 5 widgets, how long would it take 100 machines to make 100 widgets? *(1 min correct answer)*

>A bat and a ball cost $110 in total. The bat costs $100 more than the ball. How much does the ball cost? *($ 5 correct answer)*

>In a lake, there is a patch of lily pads. Every day, the patch doubles in size. If it takes 48 days for the patch to cover the entire lake, how long would it take for the patch to cover half of the lake?

*(47 correct answer)*
